# Bis(ethyl­eneglycolato-κ^2^ *O*,*O*′)tellurium(IV)

**DOI:** 10.1107/S1600536813015687

**Published:** 2013-06-12

**Authors:** Neil R. Brooks, Minxian Wu, Luc Van Meervelt, Koen Binnemans, Jan Fransaer

**Affiliations:** a KU Leuven–Universtiy of Leuven, Department of Chemistry, Celestijnenlaan 200F, B-3001 Leuven, Belgium; b KU Leuven–Universtiy of Leuven, Department of Metallurgy and Materials Engineering, Kasteelpark Arenberg 44, B-3001 Leuven, Belgium

## Abstract

The title compound, C_4_H_8_O_4_Te, crystallized from a solution of Te^4+^ in ethyl­ene glycol. The Te^IV^ atom is in a distorted seesaw coordination defined by four O atoms from two different ethyl­eneglycate ligands. The C atoms of the ethyl­eneglycate ligands are disorderd over two positions, with population parameters of 50.3 (6) and 49.7 (6)% indicating a statistical distribution. Due to the possibility to transform the primitive monoclinic unit cell into a metrically ortho­rhom­bic *C* unit cell, the data are twinned and were refined with the twin law -100/0-10/101 with the relative scale factor refining to 1.82 (4)% for the minor component.

## Related literature

For the use of Te^4+^ ethyl­ene glycol solutions in electrodeposition of Te and Te compounds, see: Nguyen *et al.* (2012 ▶); Wu *et al.* (2013 ▶). For crystal structures of related four-coordinate Te^4+^ complexes with oxo ligands, see: Day & Holmes (1981 ▶); Yosef *et al.* (2007 ▶); Annan *et al.* (1992 ▶); Fleischer & Schollmeyer (2001 ▶); Betz *et al.* (2008 ▶); Lindqvist (1967 ▶).
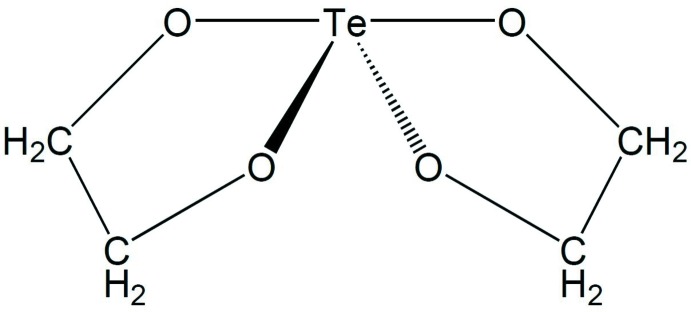


## Experimental

### 

#### Crystal data

C_4_H_8_O_4_Te
*M* *_r_* = 247.70
Monoclinic, 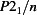

*a* = 6.4838 (7) Å
*b* = 6.4978 (8) Å
*c* = 15.3633 (15) Å
β = 102.168 (11)°
*V* = 632.72 (12) Å^3^
*Z* = 4
Mo *K*α radiation
μ = 4.64 mm^−1^
*T* = 100 K
0.20 × 0.10 × 0.08 mm

#### Data collection

Agilent SuperNova (Single source at offset, Eos) diffractometer
Absorption correction: numerical (*CrysAlis PRO*; Agilent, 2012 ▶) *T*_min_ = 0.540, *T*_max_ = 0.710
2841 measured reflections
1501 independent reflections
1291 reflections with *I* > 2σ(*I*)
*R*_int_ = 0.031

#### Refinement

*R*[*F* ^2^ > 2σ(*F* ^2^)] = 0.034
*wR*(*F* ^2^) = 0.079
*S* = 1.05
1501 reflections
96 parameters
H-atom parameters constrained
Δρ_max_ = 3.93 e Å^−3^
Δρ_min_ = −0.97 e Å^−3^

### 

Data collection: *CrysAlis PRO* (Agilent, 2012 ▶); cell refinement: *CrysAlis PRO*; data reduction: *CrysAlis PRO*; program(s) used to solve structure: *SHELXS97* (Sheldrick, 2008 ▶); program(s) used to refine structure: *SHELXL97* (Sheldrick, 2008 ▶); molecular graphics: *XP* (Sheldrick, 2008 ▶); software used to prepare material for publication: *OLEX2* (Dolomanov *et al.*, 2009 ▶) and *publCIF* (Westrip, 2010 ▶).

## Supplementary Material

Crystal structure: contains datablock(s) I, global. DOI: 10.1107/S1600536813015687/kj2224sup1.cif

Structure factors: contains datablock(s) I. DOI: 10.1107/S1600536813015687/kj2224Isup2.hkl

Additional supplementary materials:  crystallographic information; 3D view; checkCIF report

## Figures and Tables

**Table 1:** Selected bond lengths (Å)

Te1—O4	1.940 (3)
Te1—O8	1.942 (3)
Te1—O5	2.027 (3)
Te1—O1	2.032 (4)

**Fig. 1.**
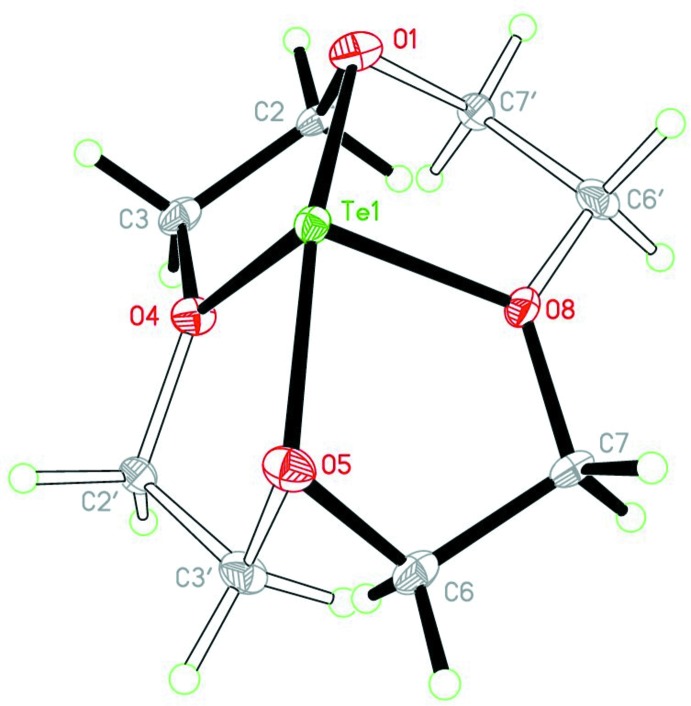
View of the asymmetric unit of Te(OCH2CH2O)2 showing the molecular structure and the disorder of the ligands. Solid bonds belong to one disorder component and open bonds to the other component (Te—O bonds are common to both components). For emphasis and to avoid confusion it should be noted that the (OCH2CH2O) ligands are disordered and the ligand is not macrocyclic.

## supplementary crystallographic information

### Comment

Solutions of Te^4+^ in ethylene glycol are interesting for the purposes of electrodeposition of Te and Te compounds (Nguyen *et al.*, 2012, Wu *et al.*, 2013). During such electrodeposition experiments we have noticed a large number of colourless crystals on the walls of the glass flask. We report here the crystal structure of these crystals.

The title compound, Te(C_2_H_4_O_2_)_2_, crystallized with one molecule in the asymmetric unit. The Te atom is in a distorted seesaw coordination defined by four O atoms from two different ethyleneglycato ligands (Fig. 1, Table 1). The carbon atoms of the ethyleneglycato ligands are disorderd over two positions, with the major component comprising 50.3 (6)% of the total. The angle between the best planes through atoms Te1—O1—C2—C3—O4 and Te1—O5—C6—C7—O8 is 85.4 (3)°. For the second position the angle between the best planes through Te1—O1—C7'—C6'—O8 and Te1—O4—C2'—C3'—O5 is 85.6 (3)°.

Crystal structures of seven similar four coordinate Te^4+^ complexes with oxo ligands have previously been reported by Day & Holmes (1981), Yosef *et al.* (2007), Annan *et al.* (1992), Fleischer & Schollmeyer (2001), Betz *et al.* (2008) and Lindqvist (1967), all of which have a distorted seesaw geometry of the Te centre. The geometries or the Te—O bond lengths in these structures do not differ remarkably from those in the title compound. The most closely related compound is the octamethyl derivative bis(1,1,2,2-tetramethyleneglycolato-*O*,*O*')tellurium(IV) (Day & Holmes, 1981), in which the Te—O bond lengths are, within error, the same as in the title compound. The O—Te—O bond angles that define the seesaw are 105.59° and 153.53° compared to 94.83 (14)° and 159.65 (13)° in the title compound. In tetrakis(methoxy)tellurium(IV) (Betz *et al.*, 2008), the O—Te—O bond angles are 89.99° and 171.42°.

### Experimental

Equal volumes of a 1 *M* solution of TeCl_4_ in ethylene glycol and a 4 *M* solution of AgNO_3_ in ethylene glycol were mixed together, ensuring that AgNO_3_ was in a slight excess. The resulting precipitate of AgCl was removed by filtration. Excess Ag^+^ ions in the remaining solution were removed by electrodeposition on a Pt working electrode at a constant potential of -0.1 V *versus* Ag and the solution used in electrodeposition experiments (Wu *et al.*, 2013). When the solution was left to stand for a period of two months, a large number of colourless crystals of the title compound slowly appeared on the walls of the glass flask.

### Refinement

The H atoms were included using a riding model, with C—H distances of 0.99 Å and *U*_iso_(H) = 1.2*U*_eq_(C). The disorder of the ligands was modelled with two atomic positions for each C atom and the relative occupancy of these two positions refined as a least-squares parameter. The population parameters of 50.3 (6) and 49.7 (6)% indicate a statistical distribution. Constraints were applied to the displacement parameters of the C atoms to keep the respective atoms from the two disorder components the same. The unit-cell dimensions are such that it is possible to transform the primitive monoclinic unit cell into a metrically orthorhombic *C* unit cell, however the high *R*_int_ for the higher Laue class shows that the Laue symmetry of the data is unequivocally monoclinic. There is however slight twinning due to these metrics and the data were refined with the twin law 1 0 0 / 0 1 0 / 1 0 1 with the relative scale factor refining to 1.82 (4)% for the minor component.. At the end of the refinement there was a residual difference electron density peak of 3.93 e Å^-3^, which was located close to the Te atom. Although careful consideration was given to the unit cell determination and the absorption correction, this peak could not be eliminated.

### Crystal data

**Table:**

C_4_H_8_O_4_Te	*F*(000) = 464
*M**_r_* = 247.70	*D*_x_ = 2.600 Mg m^−^^3^
Monoclinic, *P*2_1_/*n*	Mo *K*α radiation, λ = 0.71073 Å
*a* = 6.4838 (7) Å	Cell parameters from 1495 reflections
*b* = 6.4978 (8) Å	θ = 2.8–29.0°
*c* = 15.3633 (15) Å	µ = 4.64 mm^−^^1^
β = 102.168 (11)°	*T* = 100 K
*V* = 632.72 (12) Å^3^	Block, colourless
*Z* = 4	0.20 × 0.10 × 0.08 mm

### Data collection

**Table:**

Agilent SuperNova (Single source at offset, Eos) diffractometer	1501 independent reflections
Radiation source: SuperNova (Mo) X-ray Source	1291 reflections with *I* > 2σ(*I*)
Mirror monochromator	*R*_int_ = 0.031
Detector resolution: 15.9631 pixels mm^-1^	θ_max_ = 28.9°, θ_min_ = 3.1°
ω scans	*h* = −8→7
Absorption correction: numerical (*CrysAlis PRO*; Agilent, 2012)	*k* = −8→8
*T*_min_ = 0.540, *T*_max_ = 0.710	*l* = −20→18
2841 measured reflections	

### Refinement

**Table:**

Refinement on *F*^2^	Primary atom site location: structure-invariant direct methods
Least-squares matrix: full	Secondary atom site location: difference Fourier map
*R*[*F*^2^ > 2σ(*F*^2^)] = 0.034	Hydrogen site location: inferred from neighbouring sites
*wR*(*F*^2^) = 0.079	H-atom parameters constrained
*S* = 1.05	*w* = 1/[σ^2^(*F*_o_^2^) + (0.034*P*)^2^] where *P* = (*F*_o_^2^ + 2*F*_c_^2^)/3
1501 reflections	(Δ/σ)_max_ = 0.001
96 parameters	Δρ_max_ = 3.93 e Å^−^^3^
0 restraints	Δρ_min_ = −0.97 e Å^−^^3^

### Special details

**Table:**

Geometry. All e.s.d.'s (except the e.s.d. in the dihedral angle between two l.s. planes) are estimated using the full covariance matrix. The cell e.s.d.'s are taken into account individually in the estimation of e.s.d.'s in distances, angles and torsion angles; correlations between e.s.d.'s in cell parameters are only used when they are defined by crystal symmetry. An approximate (isotropic) treatment of cell e.s.d.'s is used for estimating e.s.d.'s involving l.s. planes.
Refinement. Refinement of *F*^2^ against ALL reflections. The weighted *R*-factor *wR* and goodness of fit *S* are based on *F*^2^, conventional *R*-factors *R* are based on *F*, with *F* set to zero for negative *F*^2^. The threshold expression of *F*^2^ > σ(*F*^2^) is used only for calculating *R*-factors(gt) *etc*. and is not relevant to the choice of reflections for refinement. *R*-factors based on *F*^2^ are statistically about twice as large as those based on *F*, and *R*- factors based on ALL data will be even larger.

### Fractional atomic coordinates and isotropic or equivalent isotropic displacement parameters (Å^2^)

**Table:**

	*x*	*y*	*z*	*U*_iso_*/*U*_eq_	Occ. (<1)
Te1	0.47847 (4)	0.12922 (6)	0.711596 (17)	0.00914 (12)	
O1	0.2476 (5)	0.3458 (6)	0.6857 (2)	0.0152 (8)	
C2	0.1002 (15)	0.2905 (17)	0.6034 (6)	0.0116 (15)	0.499 (4)
H2A	0.1543	0.3389	0.5513	0.014*	0.499 (4)
H2B	−0.0385	0.3561	0.6016	0.014*	0.499 (4)
C3	0.0770 (15)	0.0595 (17)	0.6007 (6)	0.0132 (15)	0.499 (4)
H3A	−0.0115	0.0147	0.6424	0.016*	0.499 (4)
H3B	0.0080	0.0146	0.5399	0.016*	0.499 (4)
C2'	0.3599 (14)	−0.2166 (17)	0.5966 (6)	0.0116 (15)	0.501 (4)
H2'A	0.2925	−0.2444	0.5336	0.014*	0.501 (4)
H2'B	0.3273	−0.3326	0.6332	0.014*	0.501 (4)
C3'	0.5943 (15)	−0.1981 (18)	0.6060 (6)	0.0132 (15)	0.501 (4)
H3'A	0.6578	−0.3368	0.6067	0.016*	0.501 (4)
H3'B	0.6259	−0.1211	0.5548	0.016*	0.501 (4)
O4	0.2792 (5)	−0.0285 (6)	0.6251 (2)	0.0114 (7)	
O5	0.6852 (6)	−0.0891 (6)	0.6898 (2)	0.0139 (8)	
C6	0.7477 (16)	−0.0408 (18)	0.6043 (6)	0.0136 (15)	0.499 (4)
H6A	0.6412	−0.0956	0.5538	0.016*	0.499 (4)
H6B	0.8853	−0.1056	0.6030	0.016*	0.499 (4)
C7	0.7640 (15)	0.1902 (16)	0.5960 (6)	0.0101 (14)	0.499 (4)
H7A	0.8976	0.2397	0.6340	0.012*	0.499 (4)
H7B	0.7635	0.2280	0.5336	0.012*	0.499 (4)
C6'	0.4899 (15)	0.4712 (18)	0.5992 (6)	0.0136 (15)	0.501 (4)
H6'A	0.5056	0.5106	0.5387	0.016*	0.501 (4)
H6'B	0.5554	0.5796	0.6413	0.016*	0.501 (4)
C7'	0.2586 (15)	0.4511 (17)	0.6011 (6)	0.0101 (14)	0.501 (4)
H7'A	0.1920	0.5887	0.5987	0.012*	0.501 (4)
H7'B	0.1843	0.3694	0.5496	0.012*	0.501 (4)
O8	0.5902 (5)	0.2822 (6)	0.6232 (2)	0.0122 (7)	

### Atomic displacement parameters (Å^2^)

**Table:**

	*U*^11^	*U*^22^	*U*^33^	*U*^12^	*U*^13^	*U*^23^
Te1	0.00895 (18)	0.0106 (2)	0.00793 (17)	−0.00114 (12)	0.00182 (13)	−0.00028 (12)
O1	0.0134 (18)	0.020 (2)	0.0122 (16)	0.0023 (16)	0.0029 (14)	−0.0021 (16)
C2	0.010 (3)	0.015 (4)	0.009 (3)	0.003 (3)	0.001 (3)	0.000 (3)
C3	0.011 (3)	0.015 (4)	0.012 (3)	0.003 (3)	0.000 (3)	−0.002 (3)
C2'	0.010 (3)	0.015 (4)	0.009 (3)	0.003 (3)	0.001 (3)	0.000 (3)
C3'	0.011 (3)	0.015 (4)	0.012 (3)	0.003 (3)	0.000 (3)	−0.002 (3)
O4	0.0091 (16)	0.014 (2)	0.0099 (15)	−0.0011 (15)	−0.0006 (13)	−0.0037 (15)
O5	0.0164 (18)	0.012 (2)	0.0121 (16)	0.0035 (16)	0.0014 (14)	0.0012 (15)
C6	0.010 (3)	0.016 (4)	0.016 (3)	−0.006 (3)	0.005 (3)	−0.006 (3)
C7	0.009 (3)	0.009 (4)	0.013 (3)	0.001 (3)	0.005 (3)	0.002 (3)
C6'	0.010 (3)	0.016 (4)	0.016 (3)	−0.006 (3)	0.005 (3)	−0.006 (3)
C7'	0.009 (3)	0.009 (4)	0.013 (3)	0.001 (3)	0.005 (3)	0.002 (3)
O8	0.0137 (17)	0.011 (2)	0.0143 (16)	−0.0001 (16)	0.0077 (14)	0.0024 (16)

### Geometric parameters (Å, º)

**Table:**

Te1—O4	1.940 (3)	C3'—O5	1.478 (10)
Te1—O8	1.942 (3)	C3'—H3'A	0.9900
Te1—O5	2.027 (3)	C3'—H3'B	0.9900
Te1—O1	2.032 (4)	O5—C6	1.487 (10)
O1—C2	1.460 (10)	C6—C7	1.512 (14)
O1—C7'	1.484 (10)	C6—H6A	0.9900
C2—C3	1.509 (15)	C6—H6B	0.9900
C2—H2A	0.9900	C7—O8	1.414 (9)
C2—H2B	0.9900	C7—H7A	0.9900
C3—O4	1.407 (10)	C7—H7B	0.9900
C3—H3A	0.9900	C6'—O8	1.402 (12)
C3—H3B	0.9900	C6'—C7'	1.511 (12)
C2'—O4	1.434 (10)	C6'—H6'A	0.9900
C2'—C3'	1.500 (12)	C6'—H6'B	0.9900
C2'—H2'A	0.9900	C7'—H7'A	0.9900
C2'—H2'B	0.9900	C7'—H7'B	0.9900
			
O4—Te1—O8	94.84 (14)	C3—O4—C2'	130.1 (6)
O4—Te1—O5	83.41 (15)	C3—O4—Te1	114.6 (5)
O8—Te1—O5	83.41 (15)	C2'—O4—Te1	115.2 (4)
O4—Te1—O1	82.81 (15)	C3'—O5—C6	57.7 (6)
O8—Te1—O1	82.92 (14)	C3'—O5—Te1	108.8 (4)
O5—Te1—O1	159.66 (13)	C6—O5—Te1	108.1 (5)
C2—O1—C7'	59.9 (5)	O5—C6—C7	108.7 (7)
C2—O1—Te1	108.7 (5)	O5—C6—H6A	110.0
C7'—O1—Te1	108.7 (4)	C7—C6—H6A	110.0
O1—C2—C3	108.2 (8)	O5—C6—H6B	110.0
O1—C2—H2A	110.1	C7—C6—H6B	110.0
C3—C2—H2A	110.1	H6A—C6—H6B	108.3
O1—C2—H2B	110.1	O8—C7—C6	108.7 (7)
C3—C2—H2B	110.1	O8—C7—H7A	109.9
H2A—C2—H2B	108.4	C6—C7—H7A	109.9
O4—C3—C2	108.4 (8)	O8—C7—H7B	109.9
O4—C3—H3A	110.0	C6—C7—H7B	109.9
C2—C3—H3A	110.0	H7A—C7—H7B	108.3
O4—C3—H3B	110.0	O8—C6'—C7'	109.1 (8)
C2—C3—H3B	110.0	O8—C6'—H6'A	109.9
H3A—C3—H3B	108.4	C7'—C6'—H6'A	109.9
O4—C2'—C3'	109.2 (8)	O8—C6'—H6'B	109.9
O4—C2'—H2'A	109.8	C7'—C6'—H6'B	109.9
C3'—C2'—H2'A	109.8	H6'A—C6'—H6'B	108.3
O4—C2'—H2'B	109.8	O1—C7'—C6'	106.7 (7)
C3'—C2'—H2'B	109.8	O1—C7'—H7'A	110.4
H2'A—C2'—H2'B	108.3	C6'—C7'—H7'A	110.4
O5—C3'—C2'	109.5 (7)	O1—C7'—H7'B	110.4
O5—C3'—H3'A	109.8	C6'—C7'—H7'B	110.4
C2'—C3'—H3'A	109.8	H7'A—C7'—H7'B	108.6
O5—C3'—H3'B	109.8	C6'—O8—C7	130.3 (6)
C2'—C3'—H3'B	109.8	C6'—O8—Te1	114.3 (4)
H3'A—C3'—H3'B	108.2	C7—O8—Te1	115.4 (5)
			
O4—Te1—O1—C2	−16.1 (5)	O4—Te1—O5—C3'	17.1 (5)
O8—Te1—O1—C2	79.7 (5)	O8—Te1—O5—C3'	−78.5 (5)
O5—Te1—O1—C2	31.6 (7)	O1—Te1—O5—C3'	−30.5 (7)
O4—Te1—O1—C7'	−79.7 (5)	O4—Te1—O5—C6	78.3 (5)
O8—Te1—O1—C7'	16.1 (5)	O8—Te1—O5—C6	−17.4 (5)
O5—Te1—O1—C7'	−32.0 (7)	O1—Te1—O5—C6	30.7 (7)
C7'—O1—C2—C3	138.3 (9)	C3'—O5—C6—C7	138.1 (10)
Te1—O1—C2—C3	37.0 (7)	Te1—O5—C6—C7	36.9 (8)
O1—C2—C3—O4	−45.4 (9)	O5—C6—C7—O8	−43.0 (10)
O4—C2'—C3'—O5	40.2 (10)	C2—O1—C7'—C6'	−138.4 (10)
C2—C3—O4—C2'	−147.6 (8)	Te1—O1—C7'—C6'	−37.2 (8)
C2—C3—O4—Te1	32.9 (8)	O8—C6'—C7'—O1	46.5 (9)
C3'—C2'—O4—C3	154.0 (8)	C7'—C6'—O8—C7	148.3 (8)
C3'—C2'—O4—Te1	−26.5 (8)	C7'—C6'—O8—Te1	−34.5 (8)
O8—Te1—O4—C3	−92.2 (5)	C6—C7—O8—C6'	−153.6 (8)
O5—Te1—O4—C3	−175.0 (5)	C6—C7—O8—Te1	29.2 (9)
O1—Te1—O4—C3	−9.9 (5)	O4—Te1—O8—C6'	92.7 (5)
O8—Te1—O4—C2'	88.2 (5)	O5—Te1—O8—C6'	175.5 (5)
O5—Te1—O4—C2'	5.5 (5)	O1—Te1—O8—C6'	10.6 (5)
O1—Te1—O4—C2'	170.5 (5)	O4—Te1—O8—C7	−89.7 (5)
C2'—C3'—O5—C6	−135.5 (11)	O5—Te1—O8—C7	−6.9 (5)
C2'—C3'—O5—Te1	−35.5 (9)	O1—Te1—O8—C7	−171.8 (5)
